# Health Resource Utilisation and Disparities: an Ecological Study of Admission Patterns Across Ethnicity in England Between 2017 and 2020

**DOI:** 10.1007/s40615-022-01464-7

**Published:** 2022-12-05

**Authors:** C. M. Toal, A. J. Fowler, R. M. Pearse, Z. Puthucheary, J. R. Prowle, Y. I. Wan

**Affiliations:** 1 William Harvey Research Institute, Queen Mary University of London, London, EC1M 6BQ UK; 2 Acute Critical Care Research Unit, Royal London Hospital, Barts Health NHS Trust, London, E1 1FR UK

**Keywords:** Ethnicity, Socioeconomic deprivation, Secondary care, Health inequality

## Abstract

**Background and Aim:**

The COVID-19 pandemic highlighted adverse outcomes in Asian, Black, and ethnic minority groups. More research is required to explore underlying ethnic health inequalities. In this study, we aim to examine pre-COVID ethnic inequalities more generally through healthcare utilisation to contextualise underlying inequalities that were present before the pandemic.

**Design:**

This was an ecological study exploring all admissions to NHS hospitals in England from 2017 to 2020.

**Methods:**

The primary outcomes were admission rates within ethnic groups. Secondary outcomes included age-specific and age-standardised admission rates. Sub-analysis of admission rates across an index of multiple deprivation (IMD) deciles was also performed to contextualise the impact of socioeconomic differences amongst ethnic categories. Results were presented as a relative ratio (RR) with 95% confidence intervals.

**Results:**

Age-standardised admission rates were higher in Asian (RR 1.40 [1.38–1.41] in 2019) and Black (RR 1.37 [1.37–1.38]) and lower in Mixed groups (RR 0.91 [0.90–0.91]) relative to White. There was significant missingness or misassignment of ethnicity in NHS admissions: with 11.7% of admissions having an unknown/not-stated ethnicity assignment and ‘other’ ethnicity being significantly over-represented. Admission rates did not mirror the degree of deprivation across all ethnic categories.

**Conclusions:**

This study shows Black and Asian ethnic groups have higher admission rates compared to White across all age groups and when standardised for age. There is evidence of incomplete and misidentification of ethnicity assignment in NHS admission records, which may introduce bias to work on these datasets. Differences in admission rates across individual ethnic categories cannot solely be explained by socioeconomic status. Further work is needed to identify ethnicity-specific factors of these inequalities to allow targeted interventions at the local level.

**Supplementary Information:**

The online version contains supplementary material available at 10.1007/s40615-022-01464-7.

## Introduction

Despite increasing overall health and income status, health inequalities in England have been worsening over the last twenty years [1], with 1 in 3 premature deaths thought to be attributable to socioeconomic inequality [2]. However, compared to measures of social deprivation, determinants of ethnic health inequalities remains under-investigated and poorly understood [3, 4]. During the COVID-19 pandemic, patients from Asian, Black, and minority ethnic groups experienced higher rates of hospital admission [3, 5] and worse outcomes[6] compared to white groups.

A number of explanatory factors may account for these differences, including preexisting conditions, socioeconomic, and environmental and structural determinants of health [3, 7–10]. However, the relationship between ethnicity and health is complex, in part due to the inter-relationships between these numerous factors [3]. It is important to understand how preexisting inequalities contributed to outcome discrepancies in COVID-19.

Pre-pandemic studies showed Black, Asian, and other minority ethnic groups reported worse baseline health [7, 11, 12], lower healthcare access and less satisfaction with the services provided [13–15]. However, many of these studies focused on specific chronic diseases [3, 5, 13, 16–18], on regional populations[3, 19, 20] or on national populations prior to 2014 [13]. It was only in 2021 that the UK Office for National Statistics (ONS) first released mortality statics by ethnic group [21], which is a welcomed but overdue step forward in the evaluation of health inequalities more generally. However, there is a lack of evidence on hospital admission rates across ethnic groups immediately prior to the pandemic to allow comparison with the COVID-19 admission trends.

In this study, we aimed to explore pre-COVID-19 disparities in secondary healthcare use across ethnic groups more generally to help identify national inequalities. We envisaged this might contribute to targeted policy interventions to improve the overall health status of minority ethnic groups. We evaluated the rate of hospital admissions nationally across ethnic groups in England from publicly available NHS England Hospital Episode Statistics (HES) data from 2017 to 2020. We also carried out a prespecified sub-analysis of admission rates across deciles of the index of multiple deprivations (IMD: a measure of relative deprivation for small geographical areas based on seven key dimensions such as income, employment, and health [22]) to contextualise the impact of socioeconomic differences amongst ethnic groups.

We hypothesised that pre-COVID-19 hospital admission rates would be significantly higher for ethnic minority groups compared to the White group. Furthermore, we hypothesised that similar patterns of admission rate discrepancies between minority ethnic groups in COVID-19 were also present more generally in the pre-COVID-19 population.

## Methods

### Study Design

This was an ecological study exploring the hospital admission rates and patterns across ethnic groups in England each year, from 2017 to 2020. Sub-analysis was performed for admission rates across IMD deciles. IMD distributions within ethnic groups were also analysed to contextualise our findings.

### Data Sources

We used publicly available, open-access data from NHS Digital between April 1, 2017, and March 31, 2020, comprising aggregated national summary data on admitted patient care (APC) taken from Hospital Episode Statistics (HES) for both ethnicity [23–25] and IMD [26–28]. Data within these was divided by academic/financial year; therefore, we used the starting year as the assigned year for that data, e.g. April 2017 to March 2018 is represented as 2017 in our results. Office of National Statistics (ONS) data was used for population estimates, with the 2011 census population [29] and the 2018 IMD population [30] for ethnicity and IMD deciles, respectively. The European Standard Population was used to age-standardise our datasets[31].

### Variables

The Office of National Statistics (ONS) has highlighted that there is no true consensus on what defines an ethnic group [32]. A variety of elements such as ancestry, culture, identity, religion, language, and physical appearance may contribute. However, it is self-defined, and the concepts it includes are subjective to what is meaningful to an individual [33]. Ethnic categories and their groups in this study were defined by the sixteen (plus ‘not stated’) categories used in the NHS Digital HES datasets, which mirrors the same grouping as the ONS 2001 census [34, 35]. Analysis was performed on both individual ethnic categories and aggregated into the five higher ethnic groups, namely: Asian (‘Indian’, ‘Pakistani’, ‘Bangladeshi’, and ‘any other Asian background’); Black (‘The Caribbean’, ‘African’, and ‘any other Black background’); mixed (‘White and Black Caribbean’, ‘White and Black African’, ‘White and Asian’, and ‘any other mixed background’); other (‘Chinese’ and ‘any other ethnic group’); White (‘British’, ‘Irish’, and ‘any other White background’). Although the ONS have updated these groupings in the 2011 and 2021 censuses, the NHS and NHS Digital have not. The grouping of ethnic categories in our study reflects the NHS ethnicity data collection groupings [33]. The index of multiple deprivations is derived from seven key dimensions: income, employment, health, education, barriers to housing, services, crime, and living environment[22]. An aggregate score is calculated for each lower layer super output area (LSOA) comprised of roughly 1500 people. These are then ranked across England from the most deprived to the least deprived and segregated into deciles, with IMD decile 1 representing the most deprived and IMD decile 10 the least[22]. Admissions are defined as any inpatient episode of care with at least one overnight stay in the hospital and included those admitted via emergency, waiting list, planned or another admission method route.

Our primary outcomes were the admission rates per 100,000 population annually within each ethnic group. Secondary outcomes included mean age of admission, ‘age-specific’ admission rates, and ‘age-standardised’ admission rates within each ethnic group. Age-specific admission rates were defined as the admissions per population of each ethnic group within six defined age categories, namely: 0–24; 25–49; 50–65; 65–75; 75–85; 85 + years. Age-standardised admission rates were calculated using the European Standard Population, a theoretical population adding up to a total of 100,000 that is widely used to produce such rates[31]. Prespecified sub-analysis was performed on all outcomes across IMD deciles to help evaluate differences between ethnicity and IMD effects on admission rates.

### Data Processing

Admission rates at the population level were calculated per 100,000 population within each ethnic group or IMD decile. Age-specific admission rates were calculated as rates per 100,000 population within the defined age group. The ESP was used to calculate age-standardised rates to remove age as a confounding factor. Age-specific and standardised admission rates for each group are presented as a relative ratio (RR) to the defined baseline (White or IMD 10) to allow clear comparisons between the groups. As there were no overall or age-grouped populations for the ‘unknown’ categories, they were removed from population-dependent results. This may introduce bias into our results, which we explore further in our limitations.

### Statistics

The use of the European Standard Population[31] and relative ratios introduced an estimate for which confidence intervals (CI) have been calculated. The programming language R version 4.0.2 (R Core Team 2020), was used for all data and graphical analysis.

## Results

### Population Distributions

The total population assigned to ethnicity in the 2011 census was *n* = 53,012,456. The total population assigned to an IMD decile in the 2018 dataset was *n* = 55,977,178. The majority of the population was White (85.4%, Table 1), with Asian the second-most populous (7.1%). All minority ethnic groups had a larger proportion of young people compared to White (Fig. S1).

**Table 1:** Table showing admission rates per 100,000 population and mean age of admission within ethnicity groups and index of multiple deprivation (IMD) deciles from 2017 to 2020

Category	Populations (%)	Admission rates per 100,000			Mean age of admission		
		2017	2018	2019	2017	2018	2019
Ethnicity	*n* = *53,012,456*						
*Asian*	3,763,900 (7.1)	24,494	25,699	25,999	40.5	41.3	41.8
*Black*	1,846,614 (3.5)	24,044	25,121	25,313	41.4	42.2	42.9
*Mixed*	1,192,879 (2.3)	16,559	17,531	18,035	25.5	26.1	26.9
*Other*	927,921 (1.8)	36,830	38,419	40,459	41.4	42.1	43.1
*White*	45,281,142 (85.4)	28,379	28,978	28,723	55.9	56.1	56.6
*Unknown*	*NA*	*NA*	*NA*	*NA*	50.5	51.4	52.3
IMD	*n* = *55,977,178*						
*1 (most deprived)*	5,645,392 (10.1)	32,638	33,775	33,730	47.4	47.6	48.4
*2*	5,769,812 (10.3)	30,054	30,926	30,882	49.0	49.3	50.2
*3*	5,803,649 (10.4)	28,890	29,597	29,870	51.2	51.6	51.8
*4*	5,742,481 (10.3)	28,619	29,373	29,711	53.0	53.4	53.8
*5*	5,618,052 (10.0)	28,979	29,867	29,955	54.7	55.1	55.6
*6*	5,608,081 (10.0)	28,702	29,713	29,887	56.2	56.5	56.8
*7*	5,536,417 (9.9)	28,342	29,363	29,614	57.3	57.5	57.6
*8*	5,493,599 (9.8)	27,939	28,968	29,388	57.4	57.8	57.9
*9*	5,454,856 (9.7)	27,803	28,844	28,873	57.5	57.9	58.4
*10 (least deprived)*	5,304,839 (9.5)	26,556	27,662	27,765	57.9	58.2	59.2
*Unknown*	*NA*	*NA*	*NA*	*NA*	30.5	30.5	32.4

### Population Admission Patterns

Admission rates were similar across Asian, Black, and White groups (range 24,044 to 28,978, Table 1). Much higher admission rates were seen in the ‘other’ group (range 36,830 to 40,459, Table 1). Lower admission rates were observed in the mixed ethnicity group (range 16,559 to 18,035, Table 1). The mean age of hospital admission was significantly lower in the Asian (41.8 years in 2019), Black (42.9), mixed (26.9), and ‘other’ (42.1) groups when compared to White (56.5) across all years. For IMD, the least affluent decile had the lowest mean hospital admission age at 47.6 years, with a stepwise increase to the most affluent IMD 10. Ethnicity was ‘unknown’ in 11.7% of admissions.

Admission rates between IMD deciles displayed a consistent stepwise change, with the highest rates of admission being seen in the least affluent decile (IMD decile 1, range 32,638 to 33,730 per 100,000, Table 1) and lowest rates in the most affluent (IMD decile 10, range 26,556 to 27,765 per 100,000).

### Age-Specific Admission Rates

**Fig. 1:**
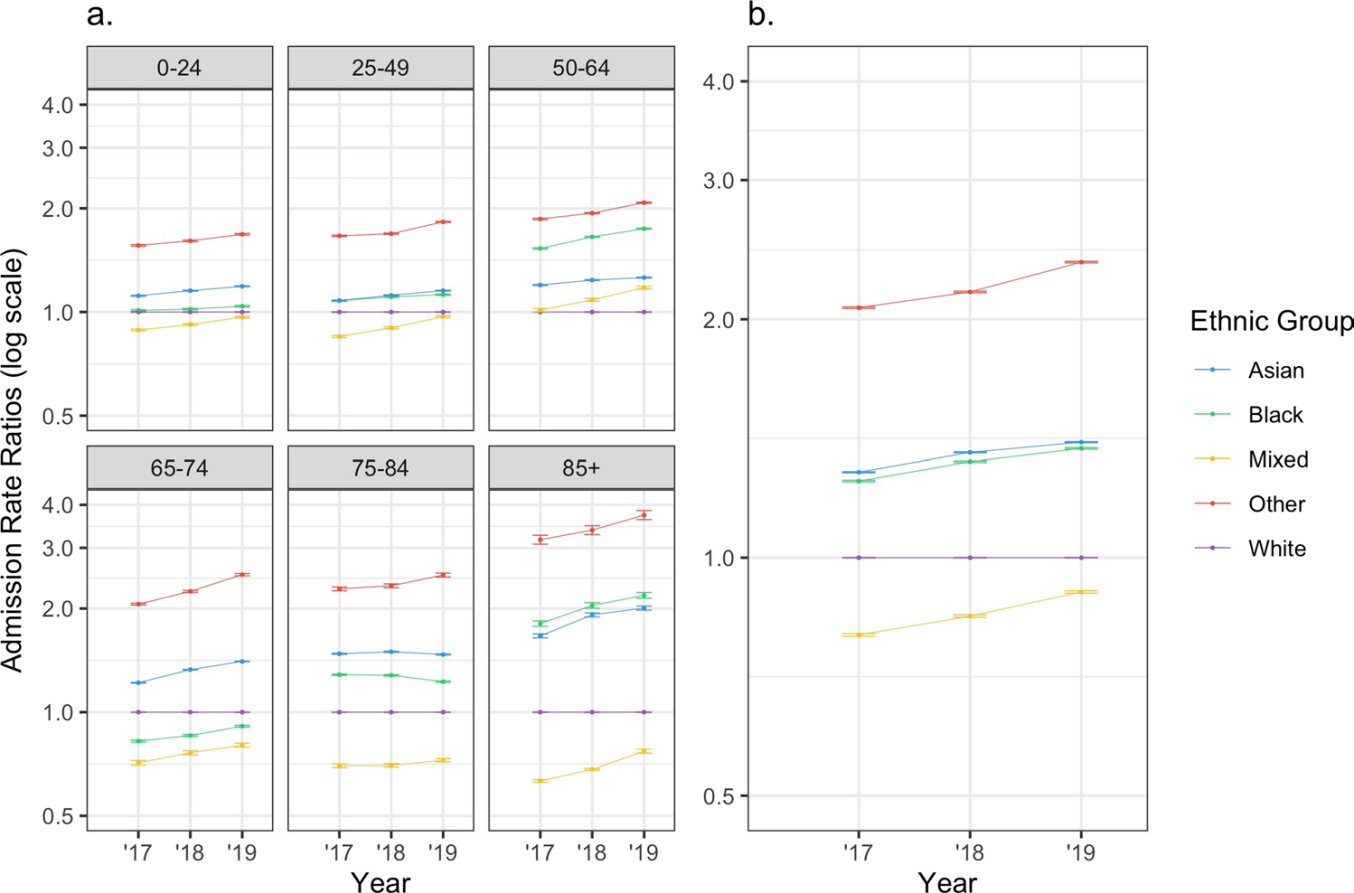
**a** Admission rates per population within ethnic groups across six different age groups, expressed as a relative ratio to a defined baseline (White population). **b** Age-standardised admission rates within ethnic groups, expressed as a relative ratio to a defined baseline (White population)

Across all age groups, Black and Asian populations showed higher rates of admission when compared to White, seen most prominently in age groups above 75 years old (Fig. 1a). Overall, the mixed ethnic population had a lower admission rate across nearly all age groups. The ‘other’ population had a significantly higher rate of admission than all other ethnicities across all age groups.

**Fig. 2:**
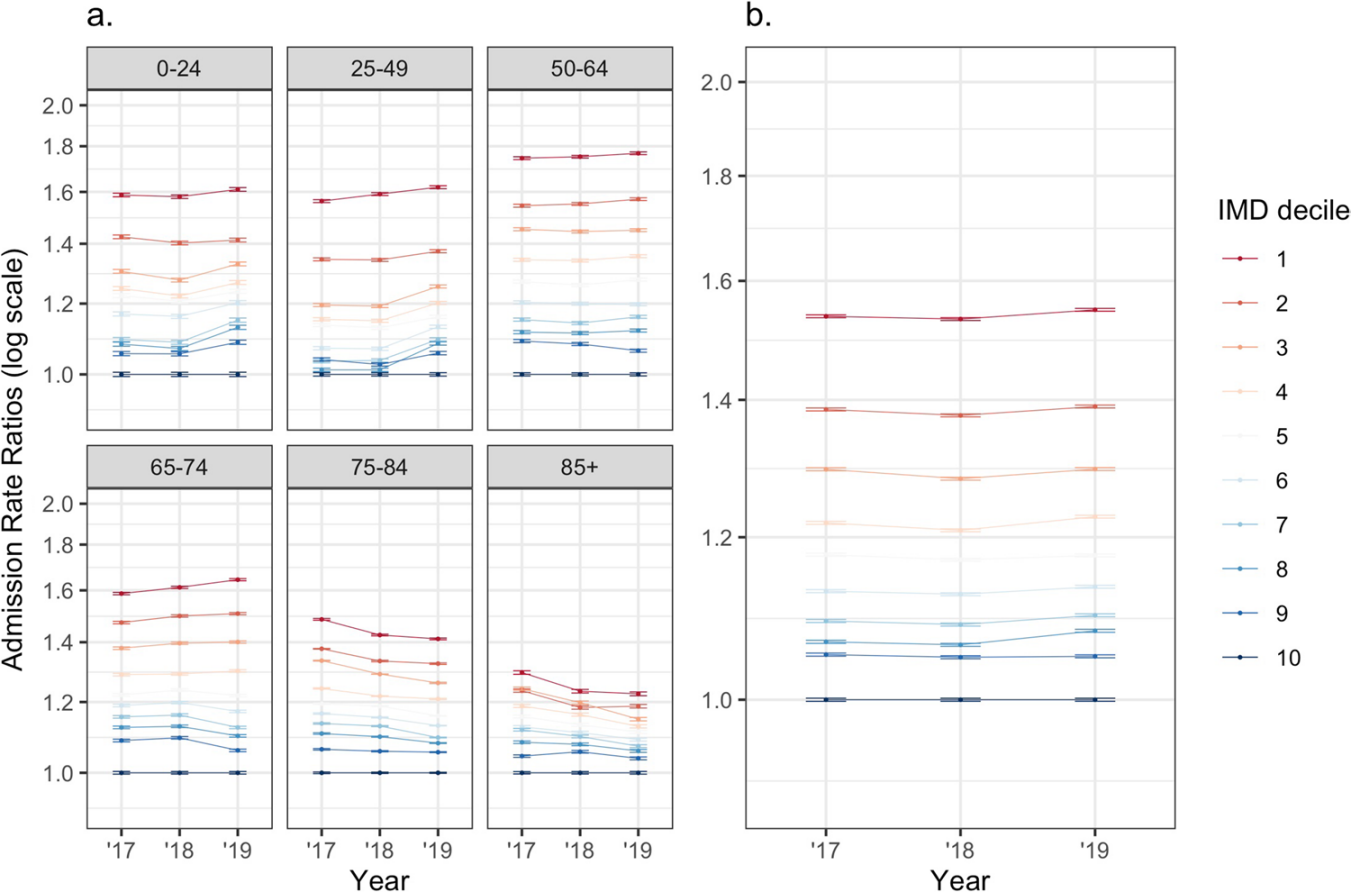
**a** Admission rates per population within IMD deciles across six different age groups, expressed as a relative ratio to a defined baseline (IMD-10). **b** Age-standardised admission rates within IMD deciles, expressed as a relative ratio to a defined baseline (IMD-10)

Admission patterns within age groups for IMD (Fig. 2a) showed a similar pattern to that seen for the total population, with increased admission rates associated with higher deciles of deprivation. However, in contrast to ethnicity, these differences were seen most prominently in the younger and middle age groups.

### Age-Standardised Admission Rates

Asian and Black populations had higher age-standardised admission rates (RR 1.40 CI 1.38–1.41 and RR 1.37 CI 1.37 − 1.38, respectively, in 2019) compared to White (Fig. 1b). The mixed population had a lower age-standardised admission rate (RR 0.91 CI 0.90–0.91 in 2019). These admission rate discrepancies were only apparent for age-specific or age-standardised rates (Table 1, Fig. 1).

The ‘other’ group showed the highest age-standardised admission rates, with over a two-fold relative rate compared to White across all years. Notably, the admission rates of all four ethnic minorities are shown to be increasing over time relative to the White population moving from 2017 to 2020 (Fig. 1b).

**Fig. 3:**
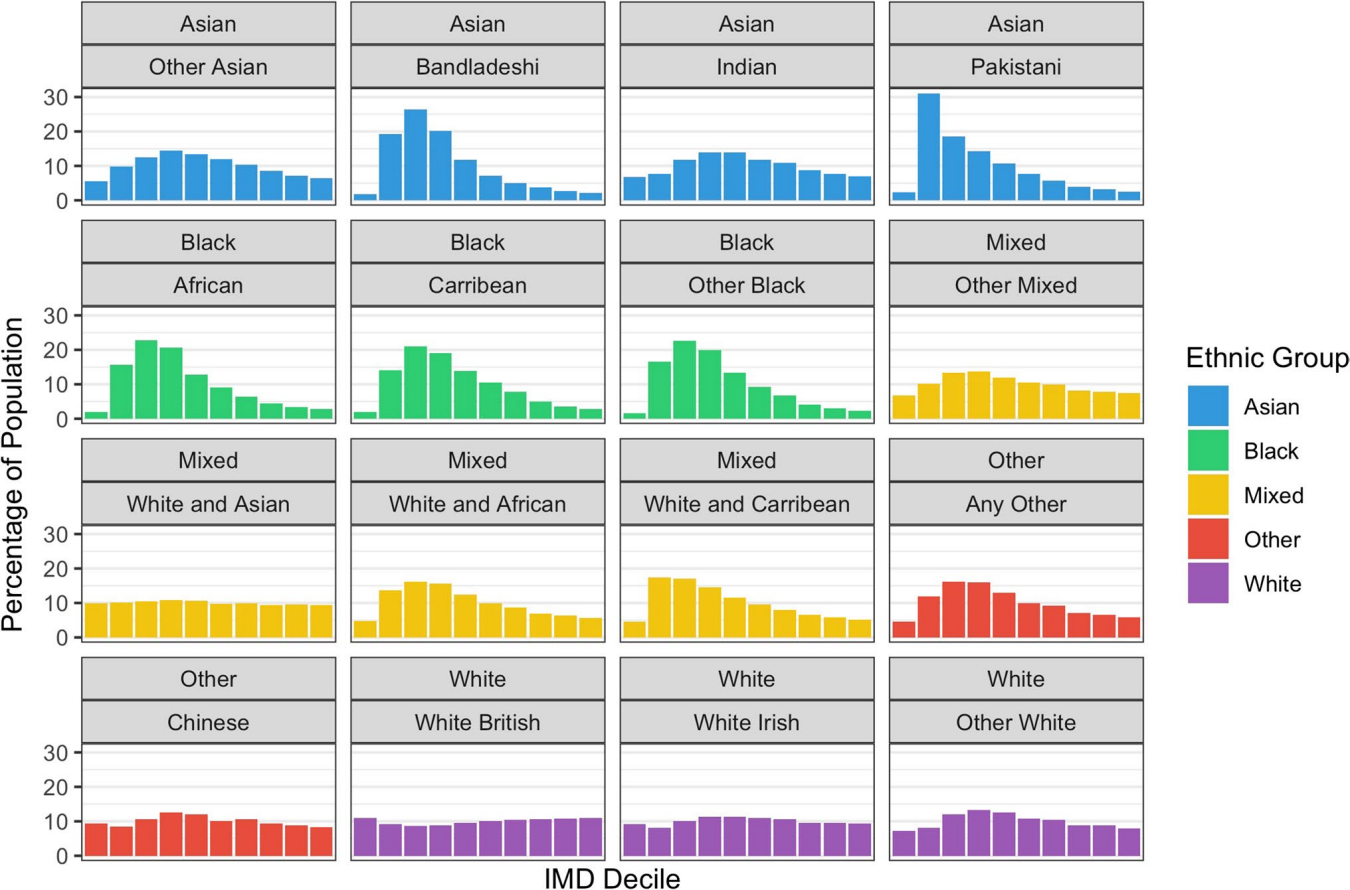
IMD distribution of ethnic populations

In addition to the main results of large ethnic groups, age-standardised admission rates were analysed for individual ethnic categories (Fig. S3). Some ethnic categories with the highest degree of deprivation, including Pakistani, Bangladeshi, Black African, and other Black, had some of the highest age-standardised admission rates. However, certain ethnic categories such as Black Caribbean, mixed White and African, and Mixed White and Caribbean had comparable or lower age-standardised admission rates compared to White British (Fig. S3). This was seen despite these minority ethnic groups having a comparably higher degree of deprivation (Fig. 3) compared to White British. Notably, within most ethnic groups, categories labelled as ‘other’ (i.e. other Black, other mixed) had the highest admission rates across most groups.

Age-standardised admission rates for IMD deciles again showed a very consistent pattern of increasing rates for more deprived deciles in a stepwise manner (Fig. 2b).

## Discussion

### Findings

The principle finding of this ecological study is Black and Asian ethnic groups show higher admission rates compared to White across all age groups and when standardised for age. There is evidence of incomplete and misidentification of ethnicity assignment in NHS admission records, as seen by the large unknown/non-stated and ‘other’ groups, respectively. This is likely to introduce significant bias to our results and all other studies using similar datasets. However, the results show differences in admission rates across individual ethnic categories cannot solely be explained by socioeconomic status.

### Findings in Context

The cause of higher admission rates in Black and Asian groups is likely linked to a number of explanatory factors that may account for these differences. Baseline health, healthcare access and prevention, discrimination, genetics, migration status and socioeconomic, and environmental and structural determinants of health may all have interrelated roles contributing to these discrepancies [3, 7–10].

It is well documented that certain chronic illnesses have a higher prevalence and manifest at a younger age in certain ethnic minority groups; for example, diabetes across all ethnic minorities, heart disease in South Asian groups (Bangladeshi and Pakistani), and hypertension and stroke in Black Caribbean and African [7]. This leads to poorer baseline health[11], higher degrees of comorbidity [12], higher disease burden [7], higher admission rates for particular conditions [3], and overall discrepancies in outcomes between ethnic groups [3, 9]. Health outcome discrepancies emerge in early adulthood and increase with age [14], with the older populations of Asian and Black groups reporting the greatest discrepancies in overall health and health-related limitations compared to White groups [36]. This is reinforced by our findings of greater admission rate discrepancies between Black and Asian groups compared to White in the older age groups.

Alongside an overall increased burden of disease, evidence suggests ethnic minorities may experience increased barriers to healthcare access and less effective healthcare provision [13–15]. It was found that ethnic minority groups may have less disease monitoring and slower intensification of therapy for certain chronic conditions [15]. Generally, they were less satisfied with the care they received [14] and reported a lower quality of care compared to White groups[14]. Worryingly, ethnic minority groups are also shown to wait longer for a medical appointment and longer to be referred to a specialist for certain conditions, including cancer [13]. Language barriers, less knowledge of available services, and discrimination may all be contributory factors [37]. Discrepancies in the timely provision of needed healthcare may contribute to worsening morbidity in ethnic minority groups. This, in turn, may exacerbate co-morbidity and complications, leading to an increased risk of acute hospital admission in the future [3, 12, 15]. Furthermore, disproportionate acute hospital presentation may reflect differences in access to healthcare in the community [3]. We did not have community data to complement our hospital admission findings, and further work is needed to compare these. Focusing policy and interventions to help remove any discriminatory or system barriers should form part of the goal to reduce these health inequalities [37].

In contrast to this, recent work on populations in Scotland [19, 20] and London [3] suggests Asians, Black, and mixed ethnicity have all-cause better survival rates after hospital admission than White. This suggests that higher admission rates of ethnic minorities may be driven by illness less associated with a high risk of death [3] despite the higher disease burden. Despite this, studies of the Scottish population [19, 20] show minority ethnic groups still have higher avoidable hospital admission, unplanned readmissions and avoidable deaths [19, 20]. Together, these highlights that all-cause mortality by ethnicity does not reflect the full picture. A further disease-specific analysis is crucial to identifying avoidable morbidity and mortality within ethnic groups.

The high incidence of ‘unknown’ and ‘not-stated’ ethnicity is a major issue for reliable analysis of this type. Guidance for the NHS and ONS states the gold standard for ethnicity recording is by self-assignment from the patient themselves rather than ascribed by someone else [38]. However, it is unclear to what extent NHS organisations are following and encouraging these principles. Previous evidence showed that, in practice, only 57% of healthcare professionals use the self-assignment method, with 21% assigning ethnicity by their own observer assessment [39]. Some studies showed only 70% of healthcare professionals routinely collected ethnicity data at all [39]. Barriers to comprehensive ethnicity data collection include lack of knowledge of staff about its importance, logistical time pressures, and lack of confidence in asking what could be perceived as sensitive information [38]. Patients themselves may also be unsure or apprehensive about how the data is used [38]. It highlights the need for training courses and protocols in the hospital to empower healthcare providers to consistently ask for and record ethnicity data. Such courses should also highlight the ways of reassuring patients about its use and importance [33]. Evidence shows that the excessive and growing numbers of unassigned ethnicity coding in NHS admissions disproportionately affect ethnic minority groups [38].

Similarly, the over-represented large ‘other’ group reflects misidentification, which is again likely to cause the underrepresentation of ethnic minorities [38]. Work by the Nuffield Trust assessed the quality of ethnicity coding in England NHS datasets and showed one-third of patients with multiple admissions had inconsistent ethnicity codes [38]. A total of 40% of those assigned ‘any other ethnic group’ also had an alternative ethnic group, with minority ethnic groups comprising two-thirds of patients impacted [38]. ‘Other’ categories within individual ethnic groups are often not accurately assigned, for example, 10% of Black Caribbean patients also had a code of ‘other Black’ in these studies[38]. Misassignment of ethnic categories within the same group hinders important conclusions between distinct populations within the same group, e.g. Bangladeshi, Indian, and other Asian. Importantly, guidance for the collection of ethnicities has not been updated in the NHS since 2001 and is no longer in line with the census categories for 2011 and 2021 [38, 40]. This presents challenges in comparing the health data across populations, and patients are not being presented with the same survey response options as those used in population estimates. The outdated classification in the NHS may also confound the selection of ‘other’ or ‘any other’ as the narrowly defined categories do not represent their ethnicity. For example, Arab, Gypsy, or Irish Traveller were not defined in the NHS categorisation but are in the more recent ONS census [40].

Linkage of hospital admission data to census-assigned ethnicity has been done by Public Health England (PHE) and other groups who have access to the individual patient data[19, 20]. This is one method of reducing ethnicity missingness or misassignment. However, the point remains that improving accuracy in ethnicity coding would allow a more robust and reliable analysis of freely accessible datasets. The differences in admission rates for Black and Asian groups were found in our study despite these factors causing underrepresentation, suggesting the differences would likely be greater if coding was more accurate.

Deducing how other factors such as genetics, migration effects, and reason for admission may impact admission rate discrepancies is not possible from the population-level data we have. However, there is widespread consensus that genetic factors contribute only marginally to ethnic inequalities, and socially constructed ethnic groups are poor markers for genetic traits, aside from specific examples such as sickle-cell anaemia [37]. A history of migration and ongoing transnational mobility can increase exposure to particular health risks [37]. Further work on more granular datasets would be useful to help identify particular disease causes that may be contributing to differential admission rates.

The strong influence of IMD on health status and outcomes is well documented [2, 41] and supported by our results of increased admission rates in more deprived deciles. It has been shown that social and economic inequalities make a substantial contribution to ethnic inequalities in health [14]. This is supported by our results showing several ethnic categories with the highest degree of deprivation, such as Bangladeshi, Pakistani, Black Caribbean, and other Black had comparably higher age-standardised admission rates. However, the different facets of socioeconomic status, such as employment, education, housing, and deprivation, all likely play different roles and exert different influences on overall health within individual ethnic categories [42]. Our results support this in the findings that specific ethnic categories (Black Caribbean, mixed White and African, and Mixed White and Caribbean) have comparable or lower admission rates than White, despite higher degrees of deprivation. One explanation for this may be ‘health resilience’, in which robust social communities within ethnic groups can shield individuals from poor health outcomes that may be associated with their degree of deprivation [43]. However, such a phenomenon would reflect partial compensation for inequalities rather than suggesting inequalities do not exist.

The finding of higher proportions of young people in the lower IMD deciles is also difficult to interpret due to the aggregate nature of the IMD marker. It may be that this is largely due to income, in which younger people who have moved out of their family home have lesser salaries compared to older generations [2]. Wealth accumulation through a person’s lifetime may contribute to these differences. However, it is possible that these findings are partially driven by those in the lowest IMD deciles living shorter lives, which has been shown in several studies [2, 14, 41].

Other studies have shown that even when admission rates are adjusted for age and deprivation, discrepancies in admission rates across ethnic groups remain [13]. This supports that ethnic inequalities in health are driven by factors other than deprivation, including overall health and health-seeking behaviours alongside discrimination and marginalisation [13–15]. Addressing socioeconomic determinants of health is necessary but not sufficient to eliminate ethnic inequalities [42]. Further work to identify the specific and unique socioeconomic pressures on different ethnic categories is required to facilitate targeting action to improve the health inequalities each group faces [37].

The establishment of the NHS Race and Health Observatory [44] represents a significant step forward to help establish health inequalities as a national priority. However, it relies on better access to high-quality data, with more accurate categorisation and protocols to improve ethnicity data imputation within healthcare systems. Effective solutions to address health inequalities require an understanding of the complexity of ethnic inequality [45]. Identifying the specific needs of different ethnic groups is imperative. Initiatives such as the introduction of integrated care systems (ICSs) represent initial steps in addressing health inequalities at the local level. These ICSs represent forty-two divided areas in the UK, allowing partnerships between NHS organisations and local authorities to facilitate the delivery of services for specific populations’ needs [46]. Presenting national discrepancies in hospital admissions and inequalities helps overall health delivery, tracking national progress over time, and comparison internationally [13, 47]. These can help frame policy at the national level, which should empower local partnerships to address region-specific differences. This could be done, for example, by allocating the necessary resources to the ICSs to enact meaningful targeted interventions to reduce preventable hospital admissions and improve outcomes in ethnic minority groups [37, 44]. However, the British Medical Association’s recent analysis [45] of the UK government’s commission on race and ethnic disparities report [48] stated that more needs to be done to ‘implement models of proportionate universalism to put proportionately more resource towards tackling the causes of worse health outcomes linked to ethnicity’. They concluded that initiatives so far have not gone far enough and ‘the structural factors that cause unlawful disparities between racial groups should not and cannot be ignored if we are to make progress.’ This highlights that there is more work to be done by the established cross-governmental committees, in partnership with local authorities, to help reduce these ongoing ethnic health inequalities.

### Strengths/Limitations

Key strengths of this work include the large and comprehensive datasets used at the population level over three consecutive years. Dividing the populations into six distinct age groups allowed reliable comparison of admission rates despite different age distributions within ethnic groups. Analysing age-standardised admission rates also removed the effects of age, which is the strongest confounding factor.

However, the data included in these large datasets represents aggregated data, which can fail to elucidate more granular differences at the individual level. Without linkage data to individual patient records, no association can be made with individual admission causes, and outcomes for ethnic groups or ameliorate coding missingness or misassignment. Headline ethnic groups were also heterogeneous and represent crude conglomerations of disparate groups, which may also introduce misrepresentation of actual background. The large ‘other’ and ‘unknown’ groups make definitive conclusions difficult.

For admissions assigned ‘unknown’ ethnicity, there is no population distribution, and it was not possible to reassign these to the correct ethnicity with the data granularity in our study. Therefore, we removed these admissions from the analysis of admission rates, which may introduce bias into our results. However, the size of these populations in HES data is an important finding in its own right, as previously discussed. Although widely used, IMD is an aggregate indicator of seven dimensions, and its use limits our ability to make interpretations about individual factors, such as income or employment. The use of the 2011 ethnicity census population was necessary as it is only released once every 10 years; however, the changes in population over this time were not captured. This also explains why there is a discrepancy in the total population numbers in the IMD-defined population (2018 dataset) and the ethnicity-defined population (2011 dataset). We do not have community data to complement our hospital admission findings, and further work is needed to compare these.

## Conclusions

This study shows Black and Asian ethnic groups have higher admission rates compared to White across all age groups and when standardised for age. There is evidence of incomplete and misidentification of ethnicity assignment in NHS admission records, which may introduce bias to work on these datasets. Differences in admission rates across individual ethnic categories cannot solely be explained by socioeconomic status. Further work is needed to identify ethnicity-specific factors of these inequalities to allow targeted interventions at the local level.

### Supplementary Information

Below is the link to the electronic supplementary material.

Supplementary Fig 1. Proportional age distribution of populations across ethnic groups (JPEG 102 KB)

Supplementary Fig 2. Proportional age distribution of populations across IMD deciles (JPEG 114 KB)

Supplementary Fig 3. Age-standardised admission rates within individual ethnic categories. Abbreviations of ethnic categories: P(A) = Pakistani; B(A) = Bangladeshi; O(A) = Other Asian; I(A) = Indian; O(B) = Other Black; A(B) = African; C(B) = Caribbean; O(M) = Other Mixed; WBA(M) White and Black African; WBC(M) = White and Black Caribbean; WA(M) = White and Asian; O(O) = Any other Ethnic Group; C(O) = Chinese; O(W) = Other White; B(W) = British; I(W) = Irish. (JPEG 294 KB)

## Data Availability

The data that support the findings of this study are openly available in NHS digital and ONS datasets. Please see references 21, 23, 24 ,25, 26 ,27, 28, 29, 30, 31 for full details to access.
