# Supplementary figures and images for: Health Resource Utilisation and Disparities: an Ecological Study of Admission Patterns Across Ethnicity in England Between 2017 and 2020
Source: J Racial Ethn Health Disparities. 2022 Dec 5;10(6):2872–81. doi: 10.1007/s40615-022-01464-7 (PMC9734479; doi:10.1007/s40615-022-01464-7)

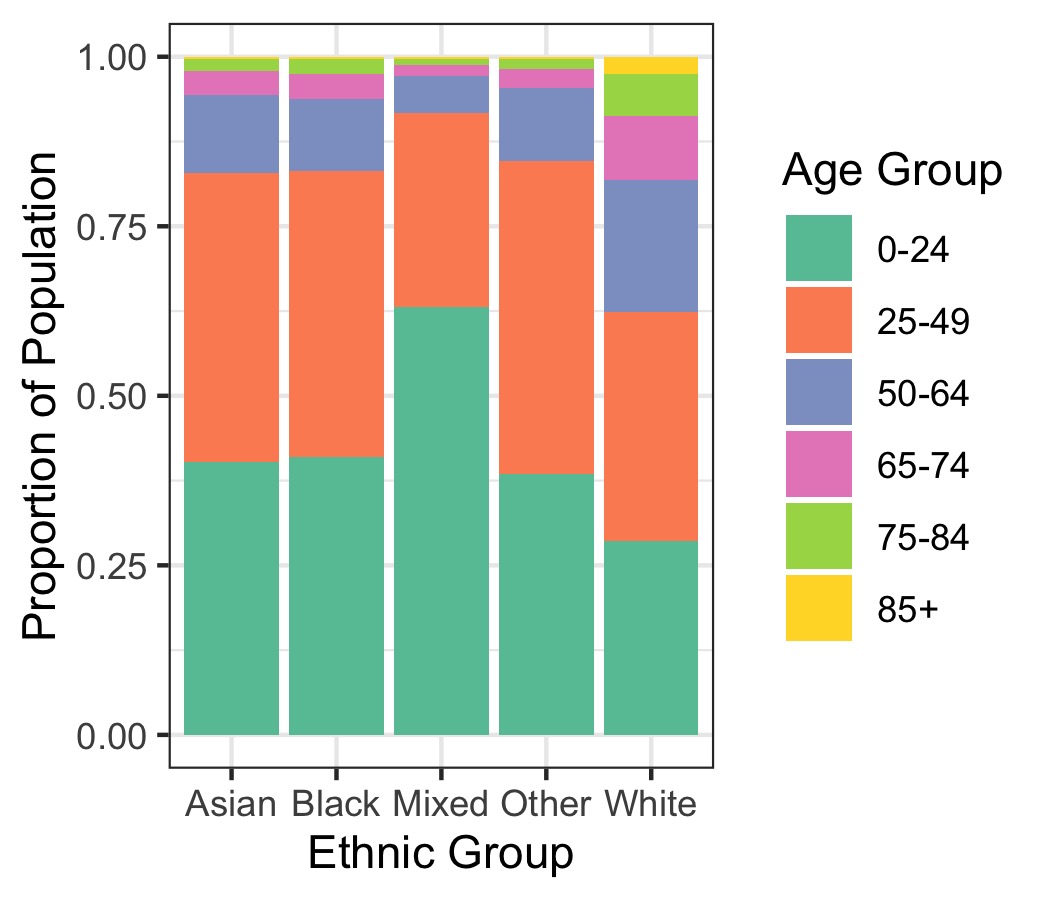

Supplement: Supplementary file 1 — Supplementary Fig 1. Proportional age distribution of populations across ethnic groups (JPEG 102 KB) [file 40615_2022_1464_MOESM1_ESM.jpeg]

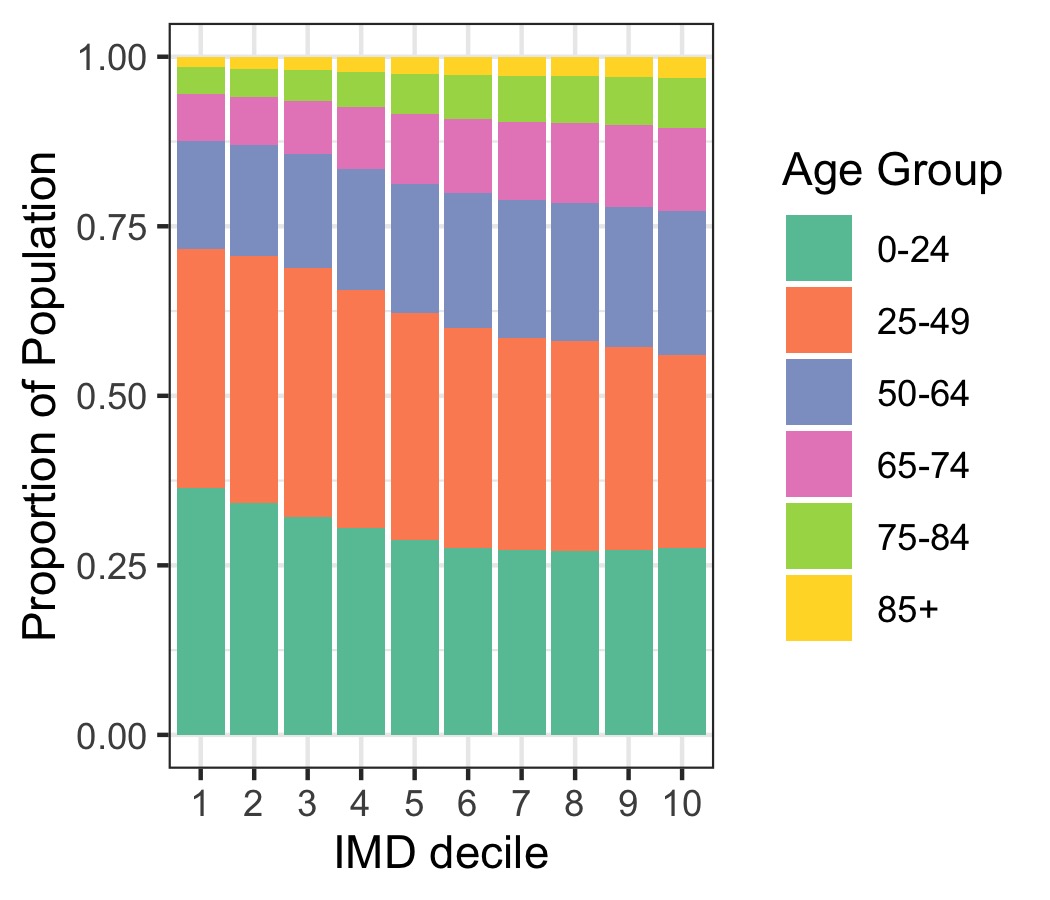

Supplement: Supplementary file 2 — Supplementary Fig 2. Proportional age distribution of populations across IMD deciles (JPEG 114 KB) [file 40615_2022_1464_MOESM2_ESM.jpeg]

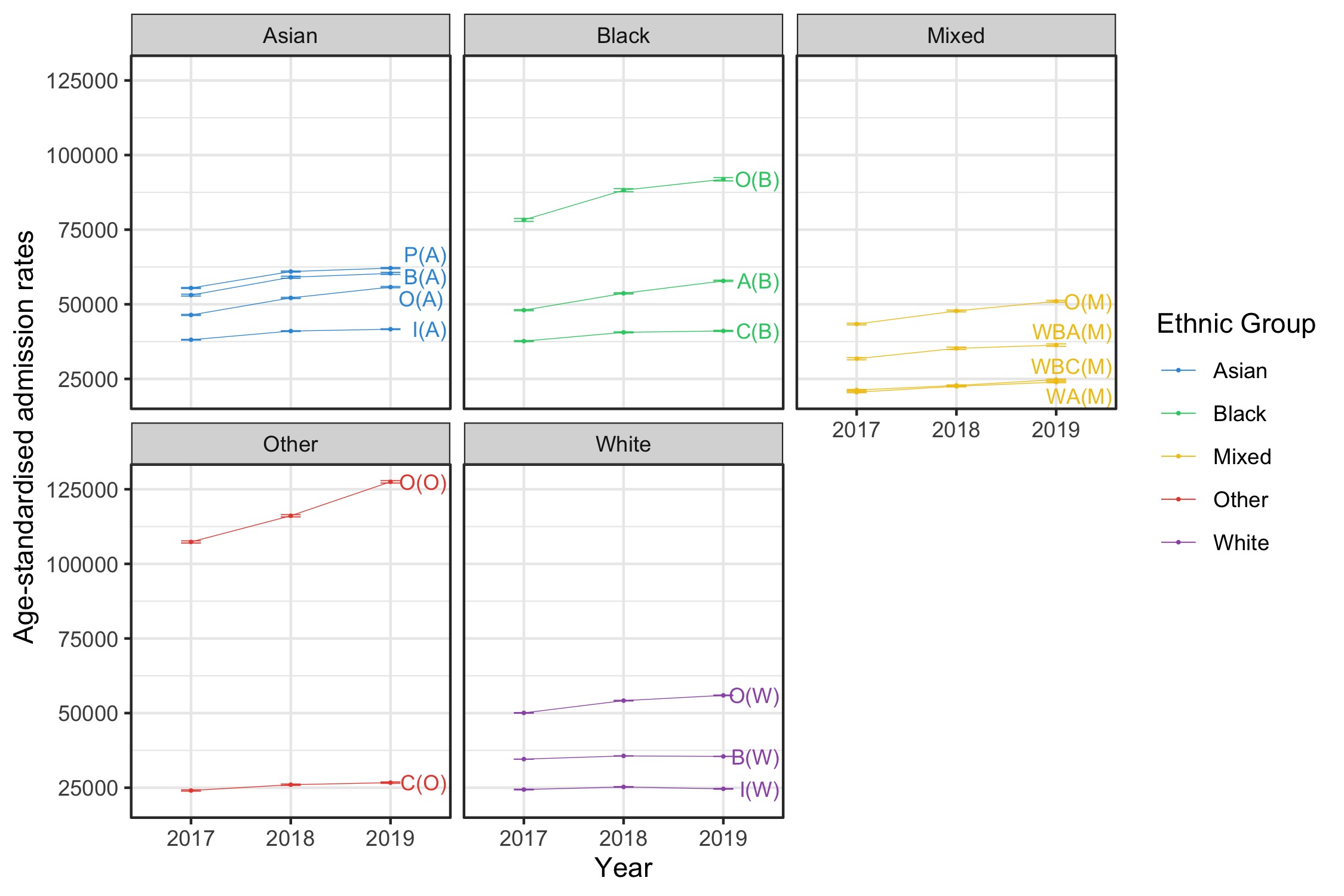

Supplement: Supplementary file 3 — Supplementary Fig 3. Age-standardised admission rates within individual ethnic categories. Abbreviations of ethnic categories: P(A) = Pakistani; B(A) = Bangladeshi; O(A) = Other Asian; I(A) = Indian; O(B) = Other Black; A(B) = African; C(B) = Caribbean; O(M) = Other Mixed; WBA(M) White and Black African; WBC(M) = White and Black Caribbean; WA(M) = White and Asian; O(O) = Any other Ethnic Group; C(O) = Chinese; O(W) = Other White; B(W) = British; I(W) = Irish. (JPEG 294 KB) [file 40615_2022_1464_MOESM3_ESM.jpeg]
